# Simulation Study of the Water Ordering Effect of the β-(1,3)-Glucan Callose Biopolymer

**DOI:** 10.1021/acs.biomac.4c01524

**Published:** 2025-02-05

**Authors:** Robinson Cortes-Huerto, Nancy C. Forero-Martinez, Pietro Ballone

**Affiliations:** † Max-Planck Institute for Polymer Research, Ackermannweg 10, 55128 Mainz, Germany; ‡ Institut für Physik, Johannes Gutenberg-Universität Mainz, Staudingerweg 9, 55128 Mainz, Germany

## Abstract

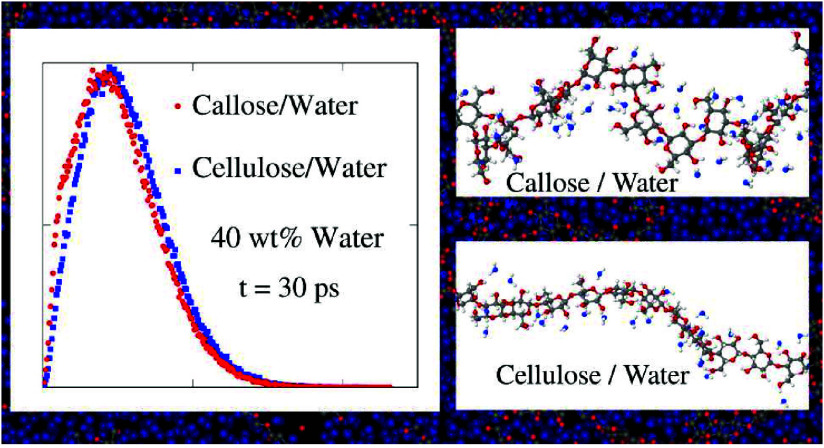

Callose, a polysaccharide closely related to cellulose, plays a crucial role in plant development and resistance to environmental stress. These functions are often attributed to the enhancement by callose of the mechanical properties of semiordered assemblies of cellulose nanofibers. A recent study, however, suggested that the enhancement of mechanical properties by callose might be due to its ability to order neighboring water molecules, resulting in the formation, up to room temperature, of solid-like water–callose domains. This hypothesis is tested by atomistic molecular dynamics simulations using *ad hoc* models consisting of callose and cellulose hydrogels. The simulation results, however, do not show significant crystallinity in the callose/water samples. Moreover, the computation of the Young’s modulus gives nearly the same result in callose/water and in cellulose/water samples, leaving callose’s ability to link cellulose nanofibers into networks as the most likely mechanism underlying the strengthening of the plant cell wall.

## I Introduction

**Figure 1:**
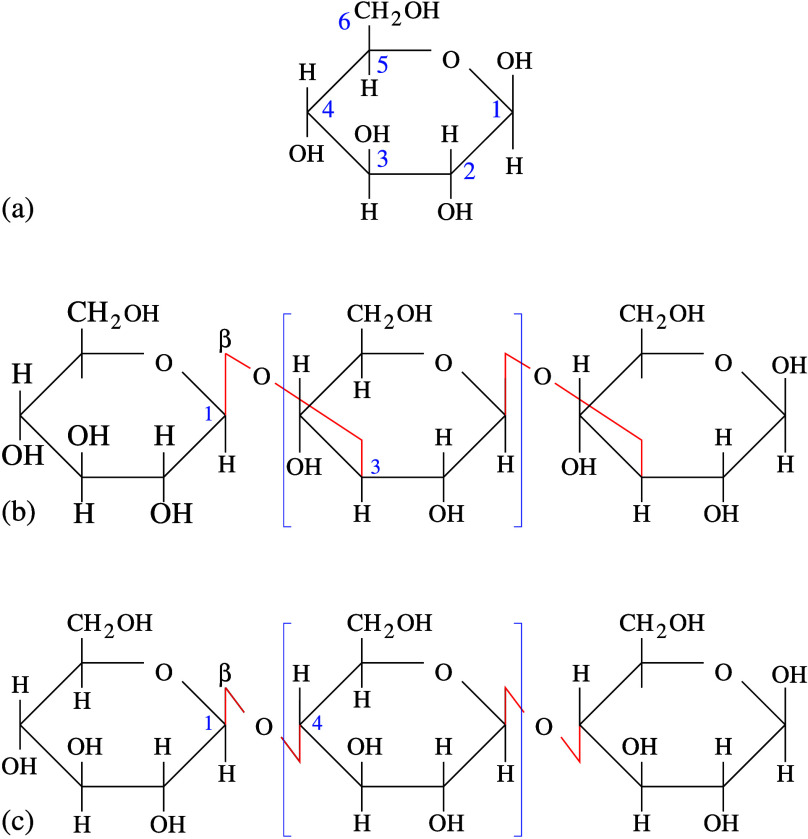
(a) Numbering of C atoms in the cyclic glucose molecule. The hydroxyl groups take the same number of the C they are bonded to. Comparison of the schematic structure of (b) callose and (c) cellulose polymers. Red line: glycosidic bonds. In (b) and (c), brackets identify the repeated unit in the biopolymers.

Callose, a paradigmatic β-(1,3)-glucan biopolymer closely related to cellulose, is a minority member of the broad family of polysaccharides that represent the major component of the plant cell wall.^1^ Depending on the origin, preparation, and context, it assumes slightly different forms of different names, with pachyman and Curdlan being the most common ones.^2,3^ The similarity of callose with cellulose is due to the fact that they display virtually the same linear primary structure (Figure 1). Moreover, at variance from other polysaccharides, such as hemicellulose and pectin, both callose and cellulose are made in the plasma membrane of plant cells, and their syntheses share their first steps.^1^ Relatively unknown for several decades after its discovery in 1863,^4^ the structure, natural synthesis pathway, and biological functions of callose have been elucidated mainly through studies of a suitable plant model, i.e., *Arabidopsis thaliana*.^5^

Despite their similarities, cellulose and callose play different roles in the plant cell wall.^6,7^ The importance of callose is emphasized by the fact that its abundance in the cell wall is finely regulated by several genes,^8,9^ and its distribution changes with changing stages in the life of the cell. Then, the accumulation of callose is promoted by callose synthase (CalS) and limited by β-(1,3)-glucanase that hydrolyzes β-(1,3)-glucosydic bonds.^10^ Variants of CalS synthesize callose that accumulates in developing pollen tubes,^11,12^ cell plates, forming along the new (double) cell wall, which divides plant cells during mitosis,^13,14^ plasmodesmata,^15,16^ sieve and companion cells,^17^ representing important elements in cell–cell transport and communication.

Besides these *normal state* functions, callose accumulates as part of the plants’ response to abiotic and biotic stress. Examples of abiotic stress eliciting callose formation are contact with toxic heavy metals,^18^ scarcity or loss of water, high temperature, or the imbalance in the concentration of primary electrolytes such as Na^+^, K^+^, and Ca^2+^.^19^ Biotic stress includes pathogenic infections by bacteria, viruses, and fungi,^20^ but may also result from the feeding of parasitic species, such as insects, worms, etc. Any attempt at penetrating the plant tissues causes the rapid mobilization (within seconds^1,21^) of CalS enzymes from neighboring cells and the deployment of callose (aptly named, in this case, *wound callose*(22)), whose aim is to plug any wound and to decrease the cell wall permeability.^23^ The healing effect, highlighted by the formation of papillae, is likely to be due to the ability of callose to join cellulose nanofibers into an elastic and impermeable network.^24^

These observations together show that callose is selectively produced by cells in the most plastic stages of their life cycle, whenever dynamic assembly of a new cell wall, rigidity, and toughness are required. While this picture emerges clearly from the biological functions of callose, the molecular mechanism underlying the observed effects is not equally apparent, not least because of the vast chemical and structural complexity of the plant cell wall.^25^ The most widely accepted interpretation points to the ability of callose to join cellulose nanofibers into extended structures (networks, gels).^24^ Support of this interpretation has been provided by chemical-physics experiments on model systems^26,27^ and also by molecular dynamics simulations of (callose–cellulose)/water gels^27^ based on atomistic models.

In the past few years, however, a different interpretation has been proposed, focusing not on the combination of callose with other polysaccharides in the cell wall, but on its interaction with water.^28^ The coincidence of an intramolecular length *d* ∼ 4.8 Å periodically repeated along the callose chain with a water–water distance in hexagonal ice could induce a crystal-like ordering in the water molecules surrounding callose in the cell wall, giving origin to a callose–water complex of remarkable stiffness and enhanced mechanical properties.

Because of the tiny size of this complex and relatively low callose concentration in most natural systems, this hypothesis, which in what follows will be named the *callose–water complex speculation*, could not be directly verified by structural and mechanical measurements. Indirect approaches could rely on the Grotthus enhancement of the proton conductivity over domains of water ordered by contact with callose. However, despite the number and variety of in vitro and in vivo set-ups suggested in ref (28), we are not aware of experimental data or unambiguous conclusions on this property.

Computer simulation and molecular dynamics (MD), in particular, provide a different route to probe the microscopic details of callose/water systems, visualizing their structure at the atomistic level. Needless to say, the reliability and accuracy of such a description depend on the quality of the force field model. However, extensive studies by molecular dynamics of structural and dynamical properties of cellulose^29^ and other related polysaccharides have shown that current force fields are adequate to provide at least a semiquantitative description of these systems. Hence, in the present study, the structure and dynamics of a network of callose chains in water have been investigated by MD, using atomistic samples of up to 1.6 × 10^6^ atoms, exploring a wide range of callose–water concentrations and covering simulation times of several ns. As a comparison, similar samples made of cellulose chains in water have been simulated, following the same protocol adopted for callose.

The simulation results for callose/water samples provide some support for the callose–water complex speculation concerning, in particular, the regularity of the distance among hydroxyl oxygens belonging to callose and show a closer association of water with callose than cellulose. Diffusion data also show a significant slowing down of water in proximity to callose, while this effect is much reduced with cellulose. This result, in particular, is reminiscent of the “immobile water layer” in contact with callose conjectured in ref (28). Simulations, however, do not find evidence of crystal-like supramolecular complexes made of callose and water and also do not find any relevant difference in the Young’s modulus of the two polysaccharides in water. These observations lend further credibility to the opinion that callose enhances the mechanical properties of the cell wall through its interaction with other polysaccharides and cellulose in particular, thus increasing the network forming ability of the different cell-wall components and strengthening the overall cohesion and resilience of the whole assembly.

## II Model and Methods

The core of the investigation is a sequence of MD simulations of atomistic samples consisting of callose and water at different wt % concentrations. Most of the simulations have been performed at NPT conditions with *T* = 300 K and *P* = 0.1 MPa (1 bar). A portion of the study concerning the estimation of the system Young’s modulus has been carried out using MD on orthorhombic samples under uniaxial load.

To provide a comparison for the results on callose/water samples, complementary simulations have been performed on similar samples in which callose is replaced by cellulose and also on samples with an equi-composition mixture of callose and cellulose in water.

A Gromos force field has been adopted. The parametrization starts from a molecular analogue of callose and cellulose made of two glucose rings joined by a glycosidic bond and terminated by an −OH group on one side and a −H atom on the other. The atomistic geometry of these analogs, optimized by a density functional approach and plane-waves algorithm (the Quantum Espresso (QE^30^) package has been used, with the PBE exchange and correlation approximation^31^) has been given in input to the ATB web-based tool.^32^ This basic model has been assembled into the force field of the chains. Since the Gromos-54a7^33^ parametrization has been gauged on the SPC water model,^34^ the same SPC model has been used to describe water in the simulated samples. A generic force field like Gromos has been preferred over a polysaccharide-specific one (like Glycam, see the classification of force fields used in polysaccharide simulations in refs (25 and 35)) in view of a future extension of the present study to more complex systems involving, besides callose, cellulose, and chitosan, a variety of other organic and inorganic species (ions) such as those entering the composition of ionic liquids.

Simulations have been carried out using Gromacs^36^ 2019 on a Linux cluster. As described below, sample sizes ranged from 0.240 × 10^6^ to 1.630 × 10^6^ atoms, requiring from 830 to 4600 core hours per ns simulation time.

The analysis of trajectories has been carried out using custom-made programs and includes the determination of structure factors, radial distribution functions, and H-bonding properties, as well as the analysis of water mobility and the estimation of Young’s modulus of selected samples. The computation of structure factors has been used to characterize the distribution of oxygen atoms throughout the systems, applying it to three different oxygen populations (): the water oxygen atoms, the polysaccharide oxygen atoms, and the total oxygen population. In each case, the determination of the structure factor is carried out first by computing
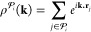
1and then computing
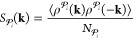
2where ⟨···⟩ indicates averaging over independent configurations and  is the number of oxygen atoms belonging to the population . The characterization of hydrogen bonding in the system is based on a purely geometric definition of H-bonds. The triplet of atoms O–H–O represents a hydrogen bond if the oxygen–oxygen distance is less than 3.2 Å, and the *OĤO* angle is wider than 140°. The relative volume to be attributed to the polysaccharide and to the water fraction in each sample was estimated using a simple MC integration coupled with a Voronoi-type definition of the volume attributed to the polysaccharide and to water. The algorithm that has been implemented is specified in Sec. SI of the Supporting Information (SI).

The mobility of water in the various samples has been analyzed by computing the self-diffusion coefficient of the water oxygen atoms and also by computing the probability distribution of distances traveled by the oxygen atoms over the relatively short time of 30 ps, as explained in Section III. The Young’s modulus *Y* has been computed using simulations in which the sample is under a uniaxial stress σ in the range 0.1 ≤ σ ≤ 10 MPa (1 to 100 bar), while the stress along the orthogonal directions is kept at 0.1 MPa (1 bar). Then, *Y* is determined from the relation between the computed relative strain [*L*(σ) – *L*_0_]/*L*_0_ (where *L* is the length of the side under stress and *L*_0_ is the value of *L* for the initial stress of 1 bar) versus σ, as described in more detail in Section III.

### II.A Sample Preparation

The precise structure of callose in the cell wall matrix of cellulose and other polysaccharides (hemicellulose) is not known; therefore, we resort to a simplified model to mimic the real system. Since water suspensions of callose tend to form gels under heating,^37^ a network-like structure of callose chains in water has been adopted as a relevant approximation of the phase of this polysaccharide in a physiological environment.

The preparation of the callose/water sample starts with the ×48 replication of a glucose ring joined by the β-(1,3) glycosidic bonds, terminated by complementary −OH and −H groups. The replication is purely geometric, thus resulting in an extended (linear) callose chain. As expected, the glycosidic bonds resulting from this geometric replication are highly strained, and a short relaxation (10 ps) is carried out by MD to reduce the high elastic energy. This short relaxation gives a slightly irregular chain but still largely linear, with an end-to-end separation of about 22 nm. Sixty-four such chains are positioned in a cubic box of 25 nm side, aligned along one side of the cube (say, *x*), going through regular positions on a square lattice in the plane of the other two sides. The origin of each chain is shifted along *x* by random distances between 0 and side *L*_*x*_ of the simulation box. Because of this random displacement, chains cross the simulation cell boundaries, which, therefore, do not represent surfaces of discontinuity for the sample.

The same chain insertion procedure is repeated with chains aligned along the other two sides (e.g., *y* and *z*), each time adding 64 chains for a total of 192 callose chains, corresponding to 9216 glucose rings. The 8 × 8 lattice for the insertion along successive directions is slightly shifted in the plane orthogonal to the chains in such a way as to avoid close contacts between the atoms belonging to different chains. The insertion of chains into the box is followed by a second short (100 ps) relaxation, intended to preliminarly relax highly strained configurations. In a further step, the box is filled with water using the *solvate* utility of Gromacs, which adds 478307 water molecules, resulting in a sample of about 1630000 atoms. The relative composition of this first sample is 85:15 wt % in water and callose, respectively.

**Figure 2:**
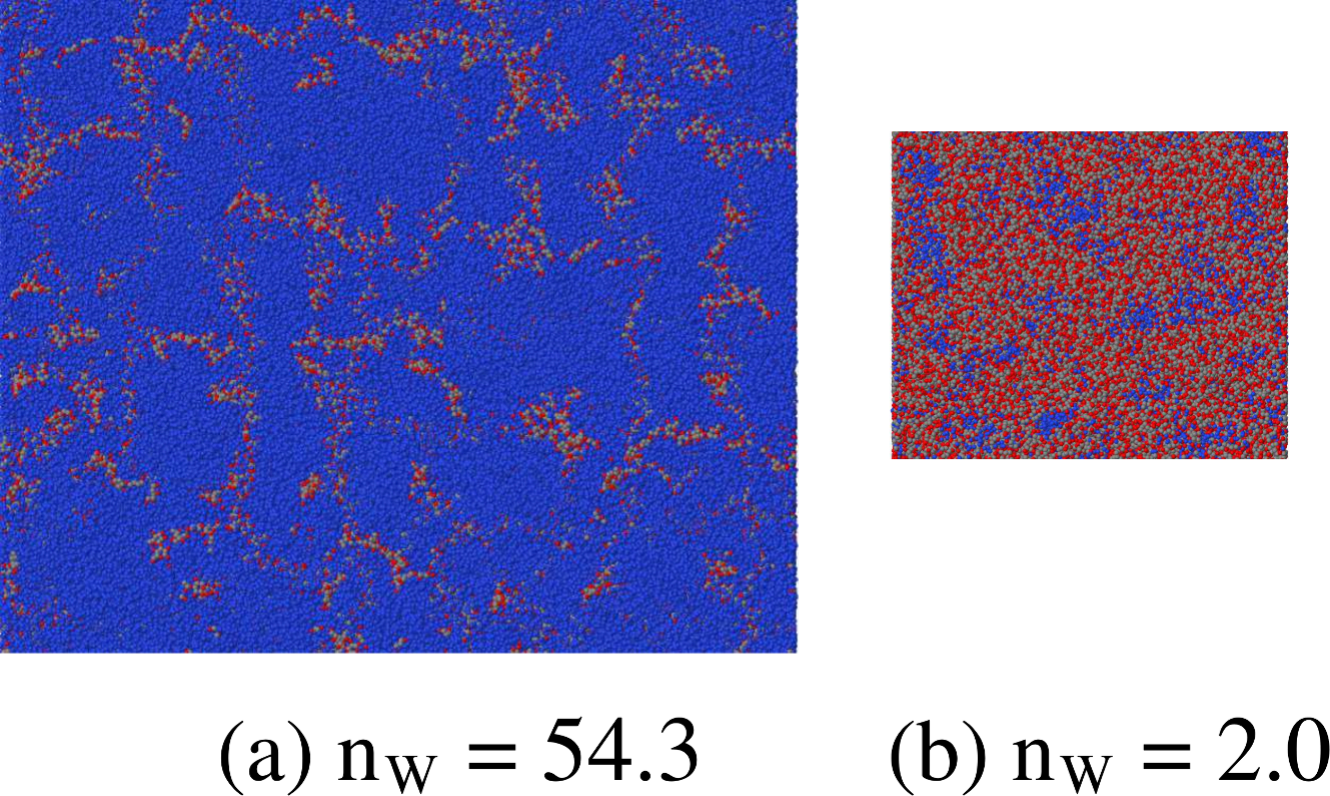
Snapshot of (a) the most dilute and (b) the most concentrated dispersion of callose in water. The two samples consist of the same number (9216) of glucose rings. (a) contains 51.9 water molecules per glucose ring and (b) contains 2.0 water molecules per glucose ring. Blue dots: O in water; red dots: O in callose; gray atoms: C in callose. Hydrogen atoms are not shown.

**Figure 3:**
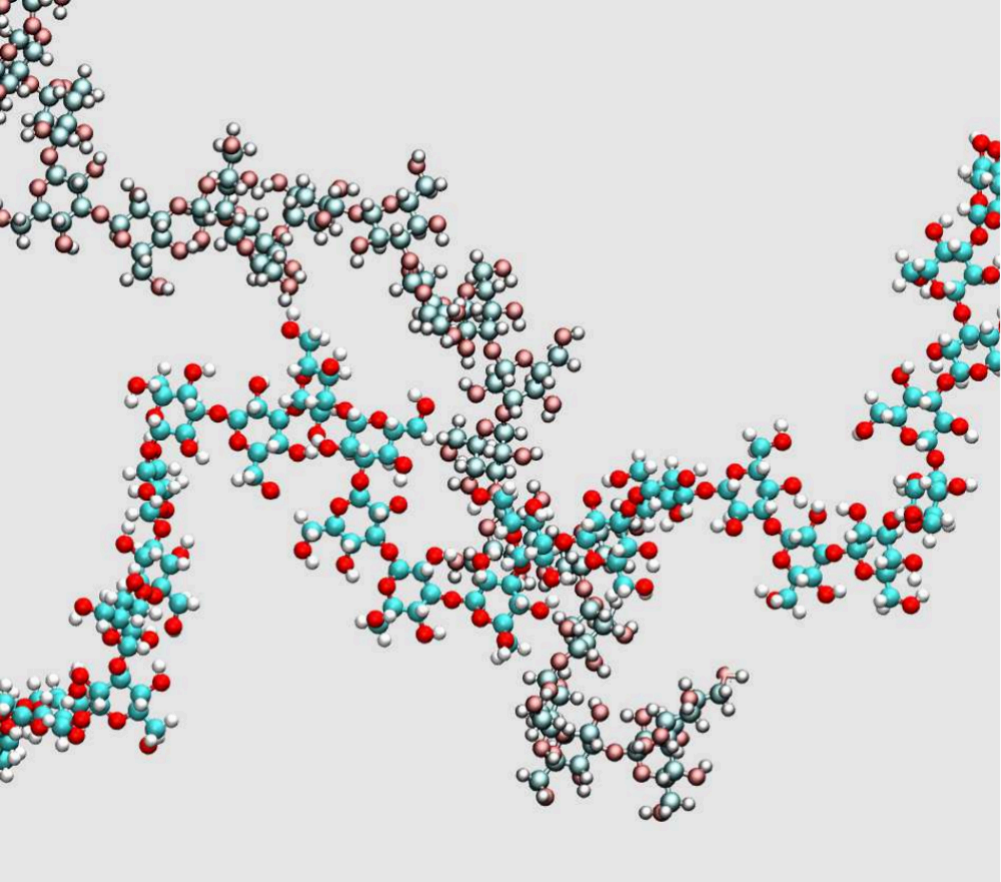
Close-contact between two callose chains in water, at relative concentration 85:15 callose/water wt %. To distinguish the two chains, all atom species were painted slightly differently on different chains.

The last stage of sample preparation consists of a 1 ns equilibration. At the end, the sample appears to be relatively homogeneous and approximately isotropic, although the chains are still largely aligned with the Cartesian axes. More precisely, the sample, shown in Figure 2a, could be described as a somewhat disordered cubic mesh of polymeric chains in water, whose overall structure is reminiscent of a hydrogel. During the short relaxation time, many chain–chain links are formed, as exemplified in Figure 3. Each of these intersections corresponds to a multitude of atom–atom close contacts stretching from one chain to the neighboring one. These contacts are primarily driven by dispersion interactions with the addition of only a few interchain H-bonds (see below the analysis of H-bonding throughout the system). The size of these contact areas varies significantly from case to case and also with changing water/polysaccharide relative composition, but typically each of them involves 12–20 atoms on each side of the intersection, forming on the order of 10^2^ short pair contacts (i.e., shorter than 5 Å), whose collective strength is sufficient to make the link irreversible with respect to room-temperature thermal fluctuations. This tendency of chains to interlink instead of dispersing themselves as homogeneously as possible in the water environment is partly due to the peculiar preparation method and initial geometry, but it is also an indication that the dissolution of cellulose and even callose in water is not thermodynamically very favorable.

The multiple cross-links, causing chain entanglement, block the network close to the initial configuration and prevent (or at least delay by several orders of magnitude in time) the further relaxation of the sample. As a result, at the end of the 1 ns relaxation, the potential energy and its different contributions from intra- and intermolecular interactions, as well as the sample volume, do not drift in a detectable way during further simulations covering times on the order of nanoseconds.

Needless to say, the simulated sample is very far from global equilibration, which, for systems of this size and complexity, is outside the reach of plain MD. Our investigation, however, concerns the distribution and mobility of water inside the polysaccharide mesh as well as the H-bonding network of water and the polysaccharide chains, which are already settled during the time of the local equilibration within the free energy basin determined by the sample preparation procedure.

From the first, a whole sequence of samples is obtained by progressively removing water, thus increasing the polysaccharide relative concentration. Each time, a given fraction of water molecules are removed at random, down to 82:18 wt % in callose/water, respectively, reached in 22 unequal steps. As expected, the water relaxation time increases with increasing callose concentration since the motion of water is increasingly constrained and its local environment becomes more complex. The length of the relaxation stage between the generation of successive samples is progressively increased to achieve the same condition of local equilibration as that for the first sample. Thus, at the highest callose concentration, the time needed to locally relax the residual 18 wt % of water is 5 ns. The generation of samples from the most dilute to the most concentrated one has been carried out strictly in a cascade; i.e., every new sample has been generated after completing the relaxation of the previous one, thus enhancing the overall equilibration of the simulated systems.

Because of the different densities of water and callose, the volume fraction occupied from the polysaccharide ranges from 11% for the most diluted to 77% for the most concentrated sample, thus deviating slightly from the concentration range measured by the wt %. The most dilute sample contains 51.9 water molecules for each glucose ring, while in the most callose concentrated sample, this ratio has decreased to 2.0 water molecules for each glucose ring.

Given the close relation between cellulose and callose, the natural benchmark to highlight the peculiarities of callose is represented by samples made of cellulose and water. However, in nature and in the plant cell wall in particular, cellulose is present as nanofibers, consisting of the semiordered aggregation of 18 chains.^38^ This structural difference would prevent a comparison of the two systems. To allow a *chain-wise* comparison of the two polysaccharides, ad hoc cellulose/water samples have been prepared, in which cellulose chains are not grouped in nanofibers but are distributed in water and prepared in a gel-like configuration using the same procedure already used for callose. Given the difference in structure and the randomness of a few preparation steps, the number of water molecules per glucose ring introduced by the solvate Gromacs utility is not exactly the same for cellulose/water and callose/water samples, but the two sequences are close to each other, allowing a detailed comparison of properties. Also, in this case, the relative polysaccharide/water concentration ranges from 14:86 to 81.5:18.5 wt %, corresponding again to a volume fraction of the cellulose chains going from 11% to nearly 77%, respectively.

The water relaxation times in between two successive sample generations turn out to be the same as for callose/water samples, increasing from 1 to 5 ns in going from the most dilute to the most concentrated sample. Again, after the short equilibration stage, the samples do not represent the global equilibrium state, but define a local equilibrium basin for the water molecules evolving in the virtually fixed network of cellulose chains. It might be worth emphasizing that this network of cellulose chains results from our preparation protocol, and its long-term relaxation is prevented by the formation of chain–chain cross-links, which in all cases takes place well within 1 ns of simulation time. Thus, the cellulose/water samples represent models to assess the peculiarities of callose and do not mimic the plant cell wall, in which cellulose is present as nanofibers.

The same procedure was followed again to prepare a third sequence of samples consisting of a disordered cubic network of 96 callose and 96 cellulose chains occupying alternating positions on the initial cubic mesh.

## III Results

### III.A MD Simulations in the NPT Ensemble

Each of the samples locally equilibrated according to the protocol described in Section II.A was simulated again to collect statistics over a time equal to the equilibration time for the same sample. In other words, the production run lasted 1 ns for the most diluted callose/water and cellulose/water samples, increasing to 5 ns for the most concentrated ones. A less extensive investigation has been carried out for the mixed callose–cellulose samples, whose results are intermediate between those of callose/water and cellulose/water systems, and for this reason they are not discussed in detail in what follows.

**Figure 4:**
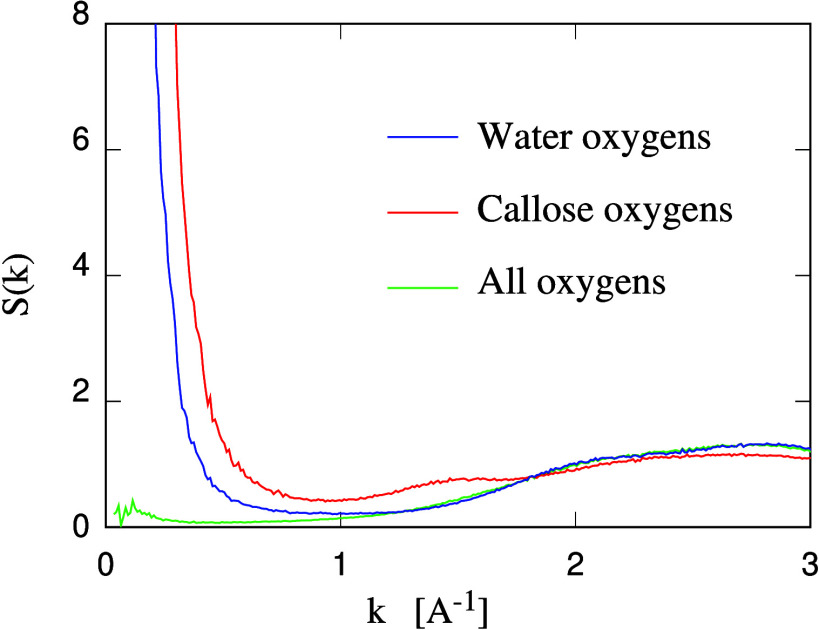
Structure factor computed for three different populations of oxygen atoms, as indicated in the labels and explained in the text. Data refer to the callose/water sample at 33.3 wt % callose concentration.

The sample preparation protocol makes systems in which the cross-linked polysaccharide chains create loose channels and interstitial domains in which water molecules diffuse. This overall structure, which can be seen by the visual inspection of snapshots, is apparent in the plot of the three structural factors that have been computed for each sample. The first of them, referring to the water oxygens, shows an apparent nanostructuring of the sample, manifested in a clear peak at *k* much smaller than the one at *k*_*nn*_ = 2π/*r*_*nn*_, where *r*_*nn*_ = 2.85 Å is the average nearest neighbor distance in bulk liquid water (see Figure 4). The *k*-vector of the prepeak, instead, corresponds to the size of the water domains, determined, as we verified, by the average separation of the callose chains. The second structure factor, referring to the oxygen atoms belonging to callose, shows a similar behavior, with a prepeak at a *k*-vector close to that of the prepeak in the first structure factor (see Figure 4). This correspondence is easy to explain, since the prepeaks reflect density fluctuations on (multi) nanometric scales, and the complementary water and callose distributions share the same approximate (space) periodicities, with an obvious interchange of the role of the filled and empty domains. The total oxygen population is significantly more uniform than each of its two parts, and the prepeak in the third structure factor is much less pronounced, being only due to the different density of oxygen atoms in callose and in water. At least for water concentration ≤36 wt %, the water–oxygen structure factor is virtually the same as the bulk water–oxygen one for *k* > 1.5 Å ^–1^ (see Figure S1 in SI).

Given these simple interpretations of the different structure factors, it is also easy to interpret the dependence of the prepeak position and shape on the water concentration (see Figure S2 in SI). In relatively dilute systems, the position of the prepeak of the water–water oxygen structure factor, in particular, moves to higher *k* with increasing callose concentration as the size and especially periodicity of the water domains shrinks. Moreover, the height of the prepeak tends to increase (again with increasing callose concentration) since the polymers’ network becomes more regular and fluctuates less. At low water concentration, however, these trends break down; since the water and polysaccharide phases are less clearly separated, water domains are less easy to identify and their distribution across the system becomes less regular. Similar considerations, with only minor changes, apply to the structure factor of oxygen atoms belonging to callose.

The oxygen structure factors for the cellulose/water and mixed callose–cellulose/water samples are very similar to the callose ones since they reflect the same geometry and qualitative bonding mechanisms in all the cases.

In the present context, the most relevant result is that, beyond ∼1.5 Å ^–1^, the total oxygen *S*(*k*) of the callose/water samples is the same as that for the cellulose/water samples. If, in the callose case, the water ordering effect is present, it does not have the long-range regularity that is required to affect the structure factors in a significant way. Incidentally, both of these structure factors are the same as the bulk water oxygen structure factor for *k* ≥ 1.5 Å ^–1^.

**Figure 5:**
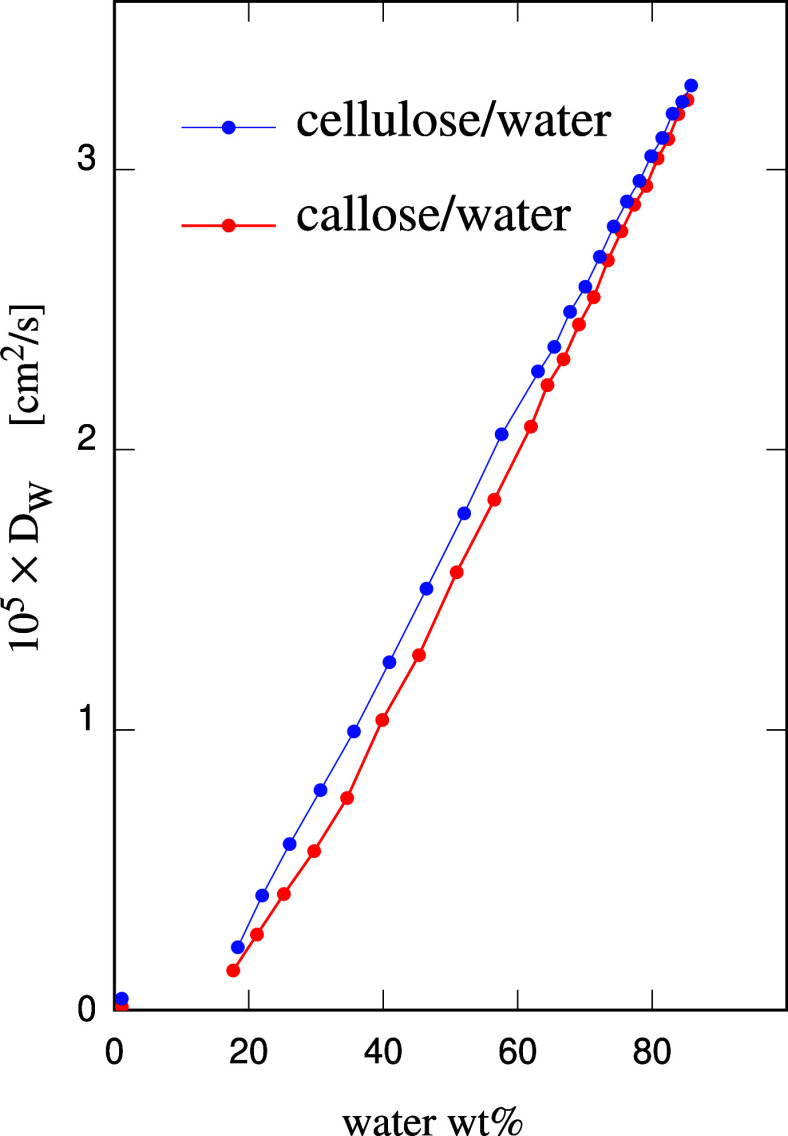
Water diffusion constant *D*_W_ as a function of water concentration (in wt %) in the polysaccharide/water samples; The error bar on each *D*_W_ value is comparable to the radius of the dots. The continuous lines are a guide to the eye. The red and blue dots close to the origin and not connected by the continuous lines represent two samples of very low water content (1 wt % water).

Because of the large mass difference, the diffusion constant of polysaccharide chains in water is orders of magnitude lower than that of water molecules. Moreover, linking chains into a 3D network reduces their mobility to values that cannot be measured by plain MD. Therefore, the only dynamic property accessible to MD concerns the mobility of water molecules in the permeable and floppy but nondiffusing polymeric gel. As a reference, bulk water simulated at equal NPT conditions using the same atomistic model (SPC water) has a diffusion constant *D*^bulk^_W_ = (4.0 ± 0.1) × 10^–5^ cm^2^/s. The computed diffusion constant *D*_W_ of water in callose/water and cellulose/water samples is reported in Figure 5 as a function of water concentration in wt %. As expected, in the hydrogel samples, *D*_W_ is significantly lower than the bulk water value. The water slowing down apparently arises from a combination of effects, including the polysaccharide volume unavailable for water diffusion, the number of H-bonds linking water and saccharide chains, and the friction due to the rapidly fluctuating, short-range interaction with the nondiffusing chains.

Despite the similarity of the two polymers and the equal number of donor and acceptor oxygens in the two cases, the difference in the diffusion coefficient is statistically significant, with the results for callose/water being systematically lower than those for cellulose/water. To improve accuracy, care has been taken to minimize the (small) errors due to the reconstruction of a continuous trajectory for every molecule, unaffected by their crossing of periodic boundaries of a simulation cell whose size fluctuates in time because of the NPT conditions. To this aim, we used the *toroidal view preserving scheme* suggested in ref (39) to unwrap the simulation trajectories. The statistical error bar on each of the *D*_W_ values in Figure 5 is comparable to the sizes of the symbols used in the plot. The separation of callose and cellulose data, therefore, is only slightly larger than two error bars combined. This might not be a very robust difference in terms of statistics, but the very systematic character of the separation (*D*_W_[callose] always being less than *D*_W_[cellulose]) increases the confidence in the result. The water diffusion in the mixed systems (callose + cellulose/water) is intermediate between those for callose/water and cellulose/water (see Figure S3 in SI), further supporting the reliability of the diffusion coefficient data. Again as expected, the *D*_W_ of the two system types tend to a common value in the limit of vanishing polysaccharide concentration, i.e., 100 wt % water, a trend that can be qualitatively recognized in Figure 5. The extrapolation of the simulation values to bulk water conditions shows that the common limit is equal to the bulk water *D*_W_^bulk^ value to within the error bars of the individual simulation data, combined with the uncertainty due to the extrapolation. The callose and cellulose data reported in the plot approach each other also in the opposite limit of low water content, but in this case the approach concerns the absolute values only, while their ratio shows that the mobility advantage of water in cellulose becomes relatively more important with decreasing water concentration. Moreover, the extrapolation toward low water content of the approximatively linear relation between *D*_W_ and the water wt % concentration suggests that water in the samples undergoes a drastic slowing down well before the water wt % concentration vanishes (see also Section SV and Figure S4 in SI). However, test simulations at a much lower water concentration (922 water molecules in ∼1850 nm^3^, corresponding to *n*_*W*_ = 0.1, and representing 1 wt % water concentration) show that the motion of water remains diffusive down to the lowest water concentrations (see Figure S5 in SI), with a residual *D*_W_ of water molecules in the nearly dry callose of *D*_W_ = (1.2 ± 0.3) × 10^–7^ cm^2^/s. At this concentration, water molecules are expected to diffuse independently from each other while interacting strongly with the callose amorphous substrate. The residual *D*_W_ of water in cellulose/water samples of the same relative concentration is *D*_W_ = (2.1 ± 0.3) × 10^–7^ cm^2^/s. These two values for callose and cellulose samples are added in Figure 5, where they appear as isolated points close to the origin. The apparent change of slope of *D*_W_ as a function of the water wt % concentration close to the origin might correspond to a parallel change of the role of water in the samples. At low water concentration, low-mobility water could stretch between neighboring polysaccharide chains, easing their linking (through hydrogen bonding) into a unique elastic aggregate. At higher water content, instead, increasingly mobile water molecules could ease the local sliding of the polysaccharide chains, acting as plasticizers.

Further insight into the mobility of water molecules is provided by the analysis of their displacement during a given time interval *t*. A Brownian particle in a homogeneous medium has the following probability to travel a distance *r* over the time *t*:
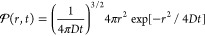
3where *D* is the diffusion constant. Discrepancies in the simulation results from this simple rule highlight deviations of the diffusion process from Brownian.

The analysis of water mobility in the callose/water samples using eq 3 has been carried out for *t* = 30 ps on the system at 60:40 wt % callose/water relative concentration. The choice of *t* and concentration has been made to optimize the signal/noise ratio in using eq 3. The choice of *t*, in particular, is determined by the presence and properties of hydrogen bonds (H-bonds) in the simulated samples, whose general features are discussed in a few paragraphs below. In this respect, the relevant information is 2-fold. First, H-bonding is one likely reason for non-Brownian dynamics in the systems under study, since the mobility of free floating water molecules will be different from that of water molecules H-bonded to the (virtually diffusionless) polysaccharide chains. This expectation is supported by recent NMR data for other polysaccharide hydrogels, i.e., alginate hydrogels,^40,41^ identifying two distinct populations of water molecules differing in their H-bonding association with the polysaccharide chains. Second, the empirical force fields of the type used in the present simulations predict a lifetime τ_*H*_ of the order of 10 ps for the H-bonds of medium/high strength typical of polysaccharide/water systems (see ref.^42,43^). Over times significantly longer than τ_H_, the interchange of H-bonded partners will blur the distinction between free-floating water molecules and those H-bonded to the polysaccharide chains, restoring Brownian statistics at the average diffusion constant of the two populations. On the other hand, for time intervals that are too short, the equilibrium fluctuation in the data can easily conceal the underlying signal. The optimal value of *t* = 30 ps in eq 3 has been found by trial and error. Concerning callose concentration, once again, one has to balance opposite effects to optimize the conditions for the analysis. In dilute systems, the relative contribution to the water mobility from molecules directly interacting with callose is too small to be distinguished from the bulk-like water background, even upon averaging over many initial configurations. At the highest callose concentration, all water molecules in the systems will be H-bonded to callose, preventing the direct comparison with free-floating water molecules in the same system. As already stated, at the 60:40 wt % callose/water relative concentration of the present analysis, the sample contains ∼55 × 10^3^ water molecules, i.e., about 6 water molecules per glucose ring. At this concentration, as apparent from snapshots, even molecules that are not H-bonded to callose cannot be considered bulk-like. However, they form nanometric pools of water occupying the interstitial spaces between the callose links, with water channels connecting neighboring pools, and, for the sake of simplicity, these molecules will be referred to as *free-floating*.

**Figure 6:**
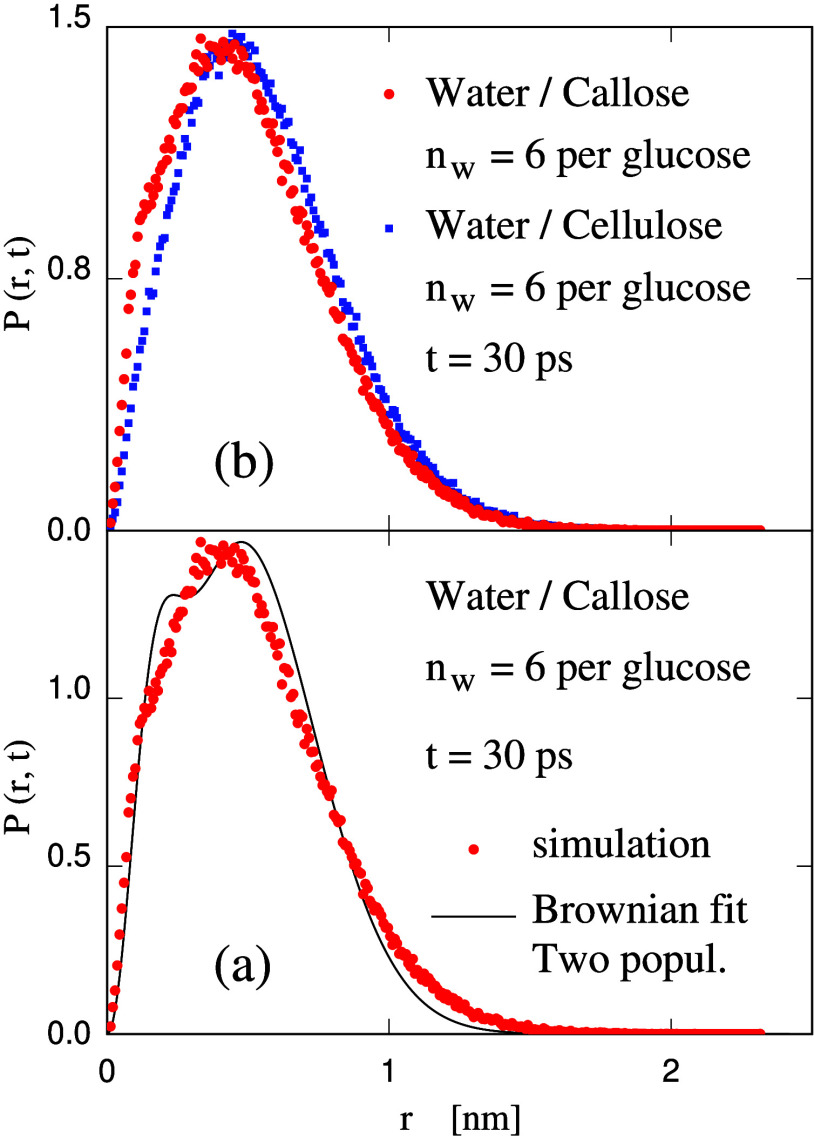
Probability distribution of water displacement over 30 ps. (a) Callose simulation data (dots) compared with the fit with two populations diffusing at different *D*_W_; (b) Comparison of the simulation data for callose/water (red dots) and cellulose/water (blue dots) samples.

After these lengthy preliminaries, the simulation results for the 60:40 wt % callose/water sample are shown in Figure 6a. No fit with a single Brownian probability distribution (eq 3) provides an accurate representation of the simulation data. The fit of lowest square deviation, in particular, underestimates the short-displacement tail (*r* < 0.4 nm) of the probability distribution obtained from simulation, as shown in Figure S6(a) in SI. The fit with two distributions, corresponding to different diffusion constants, instead is rather accurate, thus supporting the two-populations picture. According to the fit, the fast population (*D*_W_^(1)^ = 1.93 × 10^–5^ cm^2^/s) accounts for 85% of the total 55 × 10^3^ water molecules in the system, while the slow population (*D*_W_^(2)^ = 2.30 × 10^–6^ cm^2^/s) accounts for the remaining 15% of water population. Although accurate, the two-distribution fit also shows some slight residual deviations from the simulation data, possibly because the subdivision of water molecules between the two populations is broken by the short lifetime of H-bonding.

The results for cellulose/water at the same concentration are similar but quantitatively different. The detailed comparison shows that the probability distribution for the cellulose sample is biased toward higher displacements with respect to the callose sample (see Figure 6b). The fit of the simulation data with a single Brownian distribution is slightly better than in the callose case but in this case underestimates the high-displacement side of the distribution obtained from simulation, as shown in Figure S6(b) in SI. Also for cellulose/water samples, the fit with two distributions is accurate and identifies two coexisting populations, i.e., a fast one of *D*_W_^(1)^ = 2.12 × 10^–5^ cm^2^/s, accounting for 86% of the water population, and a slow one of *D*_W_^(2)^ = 4.6 × 10^–6^ cm^2^/s, accounting for 14% of the water molecules in the sample. A comparison of the results for the callose and cellulose hydrogels shows that the mobile water population is 10% faster in the cellulose network, while the slow moving population is twice as fast again in the cellulose case. It is easy to verify that the overall diffusion constant *D*_W_ is the average of fast (*D*_W_^(1)^) and slow (*D*_W_^(2)^) coefficients, weighted by the relative size of the two populations. Then, it is easy to use this relation to show that both populations contribute nearly equally to the difference in the diffusion coefficient *D*_W_ of water in callose and in cellulose displayed in Figure 5.

Looking at the displacement of individual molecules, it is easy to identify the water molecules belonging to each of the two populations. In both callose and cellulose systems and despite some ambiguous cases, it is apparent that the mobile population tends to correspond to the free floating water molecules. It is also apparent that water molecules in close contact to the polysaccharide chains belong to the slow-moving population, although a non-negligible fraction of water molecules belonging to the free floating pool (i.e., not H-bonded to the polysaccharide chains) may also display a short displacement. The two reasons that are expected to contribute the most to the slow motion of water molecules close to the (diffusionless) polysaccharide chains are the reduced volume available for diffusion and specific interactions such as H-bonds between water and polysaccharides. Unfortunately, quantifying the relative importance of these two factors is not easy.

The last observation that might be worth mentioning is that the properties of the mobile water population are different in the callose and cellulose networks. The mobility advantage of water in the cellulose over the callose network, concerning both the fast and the slow populations, intuitively suggests a hydrophilicity/hydrophobicity scale between the callose and cellulose cases, with callose being more hydrophilic than cellulose. Although we cannot prove nor quantify this relation, the conclusion fits well with what is known about the interaction of these two polysaccharides with water.

Since the ⟨|**r**(*t*) – **r**(t_0_)|^2^⟩_*t*_0__ as a function of *t* is still nonlinear at *t* = 30 ps, in both cases, the weighted sum of *D*_W_^(1)^ and *D*_W_^(2)^ somewhat overestimates the self-diffusion coefficient of water reported in Figure 5 for the same polysaccharide/water concentration.

The existence of two distinct populations of different mobility is one aspect that motivates interest in the role of hydrogen bonding between polysaccharides and water. Then, the difference between the callose/water and cellulose/water hydrogels could be explained by even moderate differences in the number, strength, and spatial distribution of these hydrogen bonds. These properties, in turn, will depend on structural features such as the accurate matching of characteristic inter- and intramolecular distances between water and polysaccharides, in qualitative agreement with the callose–-water complex speculation.

To set the stage, the number and distribution of intrachain H-bonds have been computed for all the simulated systems. The results, which represent averages over the last 400 ps of each simulation, are reported in Table I of SI. As expected, for each network type, i.e., for callose/water and cellulose/water, the number of intrachain H-bonds per glucose unit increases slowly with decreasing water content. Moreover, the number of intrachain H-bonds, always expressed per glucose unit, is comparable in the callose and cellulose case, reflecting the similarity of the structure of cellulose and callose in water as prepared according to the protocol of Section II.A. However, the number of intrachain H-bonds in cellulose/water samples slightly but systematically exceeds the number in the callose/water ones, possibly favored by the near-linearity of cellulose chains in the simulated systems, especially in the most dilute cases. The analysis of trajectories also shows that the majority of intrachain H-bonds in cellulose/water connects nearest-neighbor glucose units along the chain, while the distribution of H-bonds in callose chains is somewhat less regular. The number of H-bonds extending from a polysaccharide chain to another one is small in both cases, confirming that the close contacts among chains in the network are due primarily to dispersion interactions. As expected, the number of these interchain H-bonds increases with decreasing water content, due to the increasing proximity of chains.

**Figure 7:**
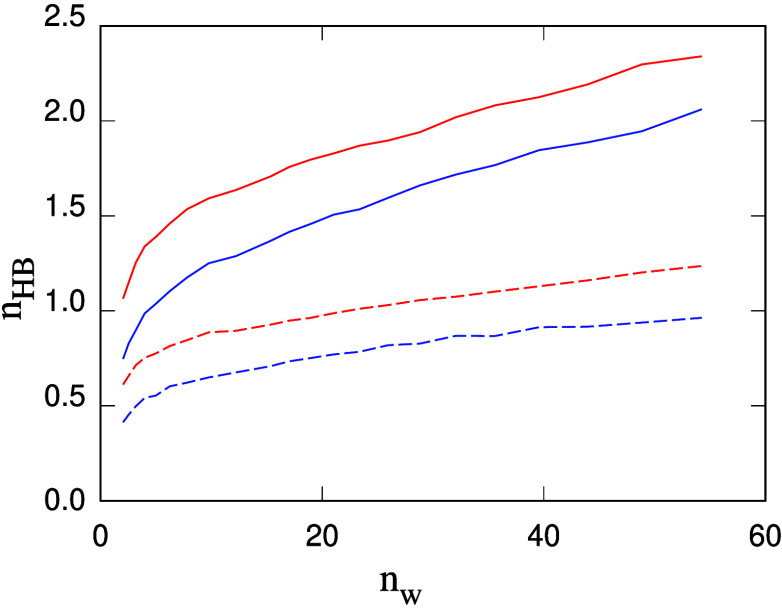
Average number *n*_HB_ of H-bonds per glucose unit for callose and cellulose chains in water as a function of the water wt % concentration in samples: red lines: callose/water; blue lines: cellulose/water; full lines: H-bonds donated by water to the polysaccharide chains; dash lines: H-bonds are donated by polysaccharides to water. Data represent averages over the last 400 ps of the production run for each system. The error bar is on the order of 2–3% of each value.

In the present context, the most relevant aspect is the number and spatial distribution of H-bonds connecting water molecules and polysaccharides. The average number *n*_HB_ of H-bonds joining water and polysaccharide chains is reported in Figure 7, divided into H-bonds donated from the saccharides to water and from the saccharides to the saccharides. Since each hydroxyl group can donate one proton and receive two, the number of H-bonds received from each glucose unit is roughly twice the number of donated bonds. Moreover, as expected, the number of saccharide-water H-bonds decreases with decreasing water content. The most apparent feature, however, is that for all systems of comparable composition and structure, the population of H-bonds is 25% higher in callose/water than in cellulose/water systems, consistent with the fact that water mobility in the former is lower than in the latter case.

**Figure 8:**
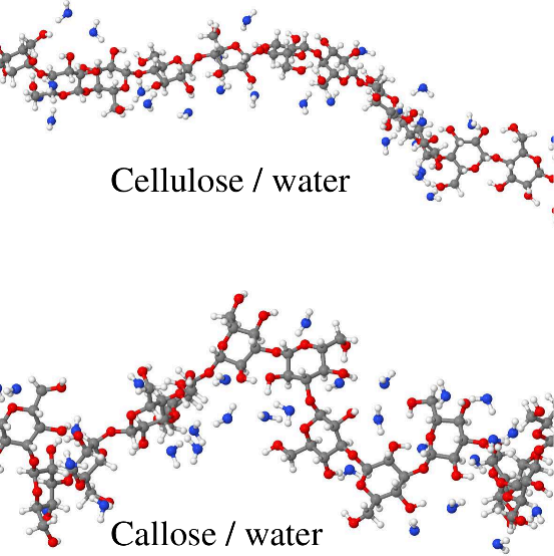
Snapshots of the most dilute (water concentration 85 wt %) callose/water and cellulose/water sample showing a segment of polysaccharide chain and the water molecules H-bonded to it. The two segments consist of nearly the same number of glucose units.

**Figure 9:**
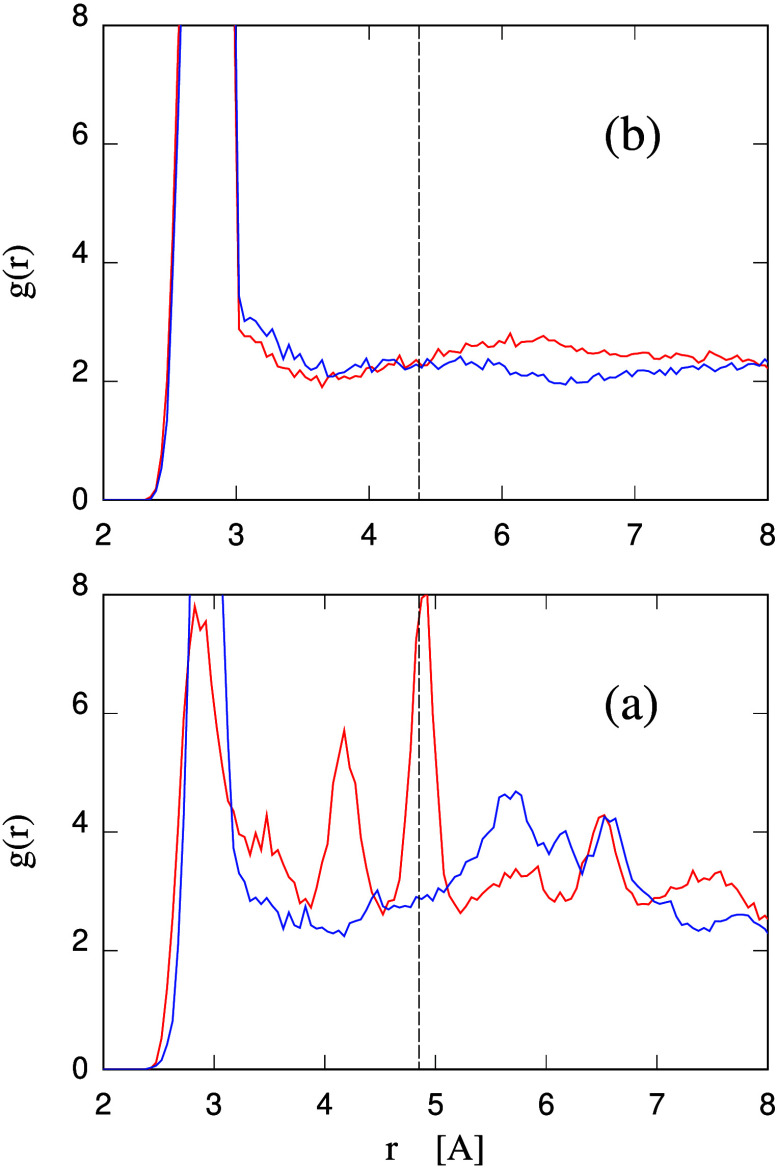
(a) Radial distribution function of hydroxyl oxygen atoms belonging to callose (red line) or cellulose (blue line) seen from a (average) hydroxyl oxygen atom of the same type at the origin. (b) Radial distribution function of oxygen atoms belonging to water molecules H-bonded to callose (red line) or cellulose (blue line) seen from an (average) hydroxyl oxygen atom belonging to the polysaccharide at the origin. All curves refer to the most dilute systems having a water concentration of 85 wt %. In both panels, the *r* = 4.85 Å radial distance is marked by a vertical dashed line (black).

A visual impression of the distribution of water molecules H-bonded to callose and cellulose is given in Figure 8. The two snapshots are representative of all systems with high water content (water concentration ≥77 wt %, i.e., *n*_W_ ≥ 30). It is apparent that despite the identical preparation and equal saccharide and water concentration, callose chains are more winding than cellulose ones. Moreover, the population of H-bonded water molecules is more abundant around callose than that around cellulose. However, even in the callose case, the water population appears to be still relatively dilute and certainly disordered (see Figure 8), contradicting the speculation of ordered, almost crystal-like water around callose. These qualitative observations are quantified by the data for the average number *n*_HB_ of H-bonds donated and accepted by water to and from callose, respectively, already displayed in Figure 7 as a function of the water content. The results show that *n*_HB_ is far from the saturation limit even in the most dilute samples, quantitatively confirming the dilute character of the population of water molecules H-bonded to callose and cellulose. A quantitative measure of disorder in the population of water oxygens H-bonded to callose and cellulose is provided primarily by the computation of radial distribution functions (rdf), that will be discussed in the next paragraph. It might be useful to anticipate that the rdf of water molecules seen by an average hydroxyl oxygen belonging to the polysaccharides (shown in Figure 9b) hardly displays any peak beyond the first one, confirming the absence of crystalline order for these water molecules. Although tall, this first narrow peak accounts for less than one H-bond donated or accepted by each hydroxyl bond of the polysaccharide to and from water, respectively. The detailed analysis of the simulation trajectories reveals several other properties. As an example, one can compute the number of water molecules accepting one H-bond from and donating one to the saccharides, thus forming chains of H-bonds along the polysaccharide. Conformations of this type could be revealed by measuring the proton conductivity, which would be enhanced by the Grotthus mechanism of proton conductivity, as suggested in ref (28). In the simulation results, however, features like this one are quantitatively not important since chains of H-bonds at the callose–water interface do not extend beyond a few units, too short to be revealed by a conductivity measurement.

As a side remark, we observe that for both callose and cellulose samples at 1 wt % water concentration, virtually every water molecule accepts/donates one or multiple H-bonds to the polysaccharide chains, while water–water H-bonds are rare. Moreover, nearly 20% of these water molecules are H-bonded to two different chains, contributing to the cohesion and elastic properties of the whole polysaccharide sample, in agreement with the picture proposed above on the complementary role of water as a cohesive (at low concentration) or plasticizer (at high concentration) component in simple polysaccharides.

As shown by the results for the H-bonding properties, the disorder in the distribution of water molecules H-bonded to the polysaccharide chains does not exclude strong correlations in their positions, which could be amplified by regularities in the structure of the polysaccharide substrate even without developing into a water crystalline phase. This picture represents, in fact, a weaker version of the callose–water complex speculation. To investigate these issues, a variety of rdfs are introduced, to be computed considering hydroxyl oxygen atoms belonging to the polysaccharides and water. Each of these functions describes the average density of oxygens of a given species as a function of radial distance *r* from an oxygen of the same or different species. To focus the analysis on the polysaccharide/water interface, the water oxygens included in the computation are only those H-bonded to the polysaccharide chains. Thus, the oxygen populations targeted by this analysis are neither homogeneous nor isotropic. Hence, the radial variable is only a convenient 1D coordinate, and the computed *g*(*r*)s do not necessarily tend to one at large distance. Despite counting oxygen atoms H-bonded to polysaccharides only, these functions are not limited to the short-range of H-bonding (*r*_*c*_ = 3.2 Å, see Section II), since they also count oxygens H-bonded to distant donor or acceptor sites on the polysaccharide chains.

The backdrop for the present discussion is provided by computation of the radial distribution function of the hydroxyl groups of callose and cellulose, shown in Figure 9a. In the callose case, the distance of the two −OH groups carried by C2 and C4 (see Figure 1a for the C atoms numbering) is *r* ∼ 4.8 Å. The −OH at C6 tends to lay parallel to the approximate plane of the glucose ring, preferentially occupying one of two positions whose distance from the hydroxyl oxygen on C4 is either *r* ∼ 3 Å or *r* ∼ 4 Å. Thus, these intraglucose pair distances account for the first three peaks in the radial distribution function of the callose hydroxyl groups. The presence of the intraring pairs in these peaks is not exclusive, since other intra- and interchain −OH pairs brought close by H-binding can provide a minority contribution to them. This is relevant especially for the peak at *r* ∼ 4.8 Å, whose correspondence with the periodicity of oxygen atoms in the hexagonal ice crystal motivated the callose-water conjecture. According to the conjecture, also the −OH groups at C2 and C4 carbons belonging to neighboring glucose rings sit preferentially at *r* ∼ 4.8 Å from each other, giving origin to long, uninterrupted sequences of callose oxygens ready to accept and donate H-bonds to water. The distance regularity along the sequence would represent an important aspect of the potential exerted by callose that could greatly enhance its ordering effect on neighboring water. The analysis of the simulation trajectories, however, shows that pairs of −OH groups at C2 and C4 carbons on neighboring glucose monomers contribute to some 15–20% (depending on water content) of the peak at *r* ∼ 4.8 Å in the radial distribution function, implying that the sequence of callose oxygens at the *r* ∼ 4.8 Å distance is divided into short segments, often no longer than two units, thus exerting a potential on water that is far from being periodic.

As a comparison, it is easy to verify that the two hydroxyl oxygens on the C2 and C3 carbon atoms of cellulose are separated by a distance of *r* ∼ 3 Å, corresponding to the first peak in the radial distribution function for cellulose −OH groups in Figure 9a. No other special distance between −OH groups can be easily seen in the ideal cellulose structure or identified in the radial distribution function of Figure 9a. Thus, the picture of −OH distances and bonding in cellulose is significantly different from the one of callose, justifying any possible difference in the effect of the polysaccharides on water (and vice versa) between the two classes of hydrogels.

Turning the attention to the water side of the callose/water system, one could observe that both liquid water^44^ and hexagonal ice^45^ (a few K degrees below the melting point) have a peak in their O–O radial distribution function at slightly different distances in the range 4.7–4.9 Å, that are associated with the formation of 6-fold molecular rings, which are stable in ice and transient in liquid water. Not surprisingly, a (weak) peak at *r* = 4.9 Å appears also in the radial distribution function computed for water molecules H-bonded to either polysaccharides (see Figure 9b), without a specific correlation with a crystalline ordering. Moreover, the *external* potential on water due to callose has indeed a component corresponding to the *r* = 4.85 Å periodicity, but this component does not appear to be particularly strong, regular or extending to long-range along the chains (see the results in previous paragraphs) to have much effect on the water structure, despite this last already displaying an inherent tendency to have a correlation peak at *r* ∼ 4.8 Å.

The radial distribution functions of water molecules H-bonded to callose or cellulose do differ (see Figure 9 b), but only at the relatively long-range *r* ≳ 6 Å, which reflects rather weakly the details of the water–polysaccharide H-bonding but is significantly affected by the different morphology of callose and cellulose networks in water and, in particular, by the meandering of callose chains. It might be worth noticing that the radial distribution functions devised to analyze the distribution of water in proximity of polysaccharides all show an excess of water oxygens at medium range (*r* ∼ 7–10 Å) from the callose chains with respect to the cellulose ones (see, for instance, Figure S7 in SI), pointing again to a somewhat higher hydrophilicity of callose with respect to cellulose, consistently with the higher number of H-bonds and stronger reduction of the water mobility already highlighted by the analyses of this section.

### B MD simulations under uniaxial stress

Besides other functions, the most likely role of callose in plants is to reinforce their cell wall in case of mechanical damage, fungi attack, and during growth stages. Although simulations do not reveal the qualitative differences conjectured in ref (28). in the properties of callose/water and cellulose/water systems (when prepared in the same way as hydrogels), it still makes sense to investigate whether callose might gain its strength from a peculiar interaction with water. To verify this point, the Young’s modulus of a relatively concentrated (60:40 wt % callose/water) sample has been computed following the approach outlined in Section II. As a comparison, the same computation has been carried out for a cellulose/water sample of the same polysaccharide concentration.

Both in the callose and cellulose cases, the simulation of stretching started from a sample equilibrated at 0.1 MPa (1 bar) during more than 2 ns. Then, the tension σ (defined here as minus the applied uniaxial stress) along one direction was increased in small steps, at first of Δσ = 0.5 MPa and then of Δσ = 1 MPa for σ > 3 MPa. At each change of tension, the sample was allowed to relax for 0.6 ns, and then the new side ⟨*L*⟩ of the simulation box along the stress direction was determined by averaging over 0.4 ns. No apparent drift of *L* was observed during the production run, following equilibration.

**Figure 10:**
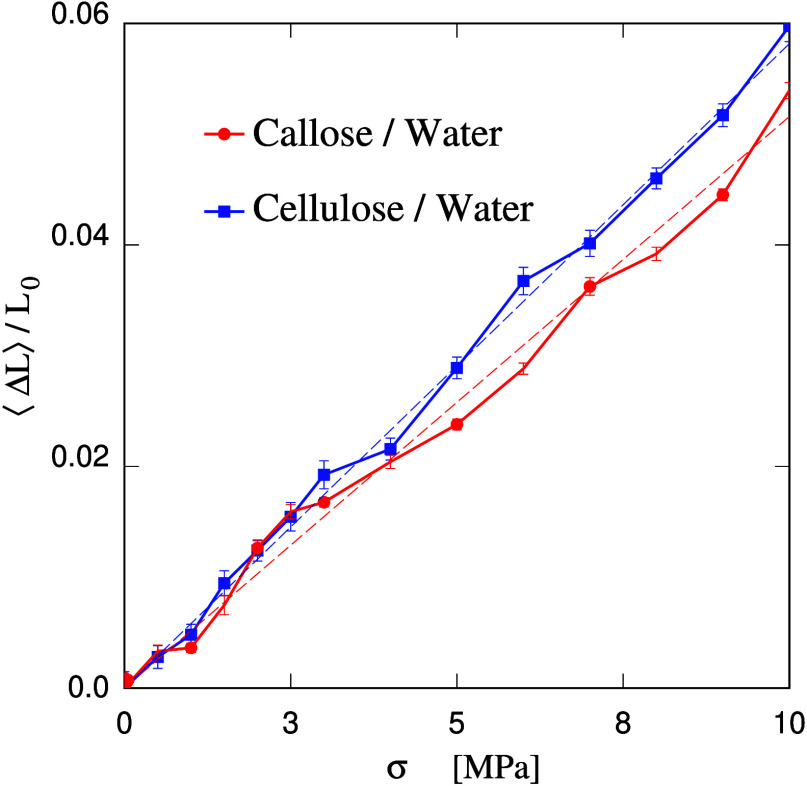
Estimate of the Young’s modulus for the callose/water and cellulose/water samples of 60:40 polysaccharide/water wt % concentration. Dash lines: linear interpolation of the two sets of data. Estimate for water/callose: *Y* = 200 ± 30 MPa; cellulose/water: *Y* = 170 ± 15 MPa

The results are shown in Figure 10. The first important observation is that both samples have a small but nonvanishing Young’s modulus, confirming the formation of a gel, since *Y* would vanish in a liquid sample made of free floating molecules. Once again, callose/water and cellulose/water samples behave slightly differently, consistently with the different linkages (β-(1,3) instead of β-(1,4)) of their glucose units. No major or qualitative difference, however, is observed, that could point to peculiar strengthening mechanisms involving the interaction of callose and water.

## IV Summary and Conclusions

Callose, a linear β-(1,3)-glucan biopolymer and a peculiar component of the plant cell wall, is selectively produced by cells in crucial stages of their life cycle. Enhanced production of callose, in particular, is a leading factor in the plants’ response to biotic and abiotic stress. These observations have long motivated the association of callose with toughness, impermeability, and overall strength of the plant cell wall.

The aim of the present study has been to investigate by computational means whether (or how much) the enhancement of mechanical properties by callose is due to its peculiar interaction with water, and, in particular, to its ability to order neighboring water molecules into an ice-like structure.^28^

To verify this hypothesis, MD simulations have been performed based on all-atom models of networks of callose chains in water. The investigation covered a wide interval of water/callose relative concentrations, involving systems of size up to 1.6 × 10^6^ atoms, and simulations reaching up to several ns.

As a comparison, the same sample preparation and simulation protocol has been applied to analogous cellulose/water systems. The comparison, however, requires some clarification, since, at normal conditions, cellulose and callose are found in different phases: callose tends to adopt an amorphous structure, while cellulose forms crystalline nanofibers that precipitate out of a water solution. To prevent this differentiation, that would spoil a meaningful comparison, the two polysaccharide/water samples are prepared in exactly the same way, putting chains on a regular 3D grid (see Section II) before relaxing them for times of the order of nanoseconds. The entanglement of chains locks the polymeric fraction in a metastable configuration, similar to the amorphous state of callose in water and prevents cellulose chains from forming nanofibrils. Because of the metastable character of both configurations, the time duration of the MD relaxation and production runs has been kept moderate but still sufficient to locally equilibrate the structure and dynamics of the water molecules.

The simulation results provide some support for callose–water complex speculation. First of all, the visual inspection of the structure and the computation of the radial distribution function for the hydroxyl oxygens belonging to callose reveals a clear peak at the distance *r* = 4.85 Å that corresponds to the periodicity of water-callose H-bonding sites that originally motivated the callose–water speculation. The same radial distribution function computed for cellulose in its metastable network configuration does not show such a correlation peak. Moreover, the diffusive dynamics of water within the callose network is significantly affected by the formation of H-bonds with the polymeric chains, while the same effect for water and cellulose is weaker and possibly reversed. This observation confirms the preferential binding of water to callose via hydrogen bonding.

The analysis of the distribution of water molecules around callose, however, does not reveal a clear layer of ice-like water. In particular, the population of water molecules H-bonded to callose turns out to be rather dilute and disordered, despite possible weak indications of incipient ordering consisting of short chains of H-bonds made by an alternation of water and callose protons and oxygens. Perhaps more importantly, there is no qualitative difference in the mechanical strength of callose and cellulose networks in water, as measured by the Young’s modulus estimated for the two system types.

Hence, the results of the present study support the traditional view that attributes the strengthening of the plant cell wall by callose to its interaction with cellulose crystalline nanofibers, as also indicated by the results of previous experiments^26^ and simulations.^27^ The tendency of callose to adopt disordered configurations consisting of single (i.e., not joined in ordered fibrils) chains provides the glue that quickly binds together cellulose nanofibers and possibly other polysaccharides into a solid-like assembly, that endows the cell wall with plasticity and toughness.

The callose–water complex speculation is the last of a series of similar hypotheses concerning the presence and role of crystal-like water in biochemical/biological systems, including sugars, steroids, triglycerides, proteins, etc., up to the complexity of biomembranes, viruses and cells, as reviewed in ref (46). These older speculations have also been generally disproved using primarily spin–echo NMR and X-ray diffraction. Similarly to the outcome of the present computations, these experimental approaches found evidence of strong H-bonding at hydrophilic water/biointerfaces, but did not confirm the existence of water molecules whose ordering extends beyond the immediate neighbors, that is instead the true signature of ice-like water systems.

Besides the results concerning the verification of the callose–water complex speculation, the present studies have many other reasons of interest in the broad context of biohydrogels, and especially, but not only, in the subtopic of computer modeling and simulation of these complex systems.

The first point to emphasize is that both callose and cellulose can be prepared in hydrogel form not only in the computational setting but also in reality, although this requires the intermediate step of dissolving them in ionic liquids, and, in the case of cellulose, a sizable fraction of nanocrystalline phase will remain (see, for instance, refs (27 and 47)). This opens the possibility of checking the computational results by experiments. This task, however, is hampered by the disordered state of these systems, complicating the use of X-ray or neutron diffraction. Other techniques, such as vibrational spectroscopy, provide a spatially averaged view of the sample properties, while direct comparison with simulation requires a local view on the microscopic scale. In this respect, a few NMR techniques could have an edge, because of their ability of determining the variety of microscopic environments surrounding the NMR-active isotopes in the sample, as well as analyzing the dynamical properties of water molecules. Investigations of this type have been carried out in the past on several hydrogels such as, for instance, paradigmatic biohydrogels such as alginates^40,41^ and chitosan-based hydrogels,^48^ and could conceivably be applied to callose and/or cellulose hydrogels. Covering the widest possible range of systems by experimental and computational approaches is crucial, since (i) experiments and computational modeling present complementary strengths and limitations, and their simultaneous application can greatly enhance their effectiveness and stimulate further developments; (ii) only the comparison of results for many different systems can highlight the role of different aspects (such as strength, structure and distribution of the links, functional groups carried by the chains, and presence and nature of ions) in determining the properties of hydrogels and predicting the phase diagram of the responsive ones, that is crucial for applications.

A significant first relation between callose, cellulose, and alginate hydrogels is already apparent in the properties investigated in the present study, represented, in particular, by the different mobility of water molecules in callose and cellulose hydrogels, that we attribute to different H-bonding strength in the two systems. This is reminiscent of the experimental identification of two distinct water populations in alginates, discussed in refs (40 and 41), and attributed again to the different H-bonding patterns and strengths between the two monomeric units that define alginate, i.e., the mannuronic and guluronic residues.

Additional reasons of interest in this study concern purely computational aspects. The complexity of hydrogels requires large samples and long simulation times as well as reliable force field models to adequately reproduce their properties. For this reason, up to now, the exploration of hydrogels by computational means has been far less extensive than desirable. The continuous and rapid growth of computer power is making routinely feasible MD simulations of 10^7^ particles over μs. Studies like the present one, involving samples of 10^6^ particles and several nanoseconds times, provide the validation of the models as well as valuable experience in the preparation of samples and data analysis required for the new wave of biohydrogel simulations that is expected to follow the coming into production of exascale-class computer facilities.

Last but not least, the full exploration of hydrogel properties will require the application of coarse-grained methods besides atomistically detailed models. To this aim, the present study provides an extended database of MD trajectories at the atomistic, explicit water level to tune coarse-grained, possibly implicit solvent models of callose and cellulose hydrogels.

## Supporting Information Available

Plots of the water diffusion coefficient in systems with mixed callose and cellulose chains; Diffusive behavior of water in concentrated callose and cellulose samples; Fits of the probability distribution of water displacements; Oxygen–oxygen radial distribution functions; Table of intrachain H-bonding abundance (PDF)
