# Supplementary material for: Simulation Study of the Water Ordering Effect of the β-(1,3)-Glucan Callose Biopolymer
Source: Biomacromolecules. 2025 Feb 5;26(3):1748–60. doi: 10.1021/acs.biomac.4c01524 (PMC11898071; doi:10.1021/acs.biomac.4c01524)
Supplement: Supplementary file 1 — bm4c01524_si_001.pdf [file bm4c01524_si_001.pdf]

A simulation study of the water ordering effect of the β-(1, 3) glucan callose biopolymer

Robinson Cortes-Huerto[1,*], Nancy C. Forero-Martinez[2] and Pietro Ballone[1]

(1) Max-Planck Institute for Polymer Research,

Ackermannweg 10, 55128 Mainz, Germany and

(2) Institut für Physik, Johannes Gutenberg-Universität Mainz,

Staudingerweg 9, 55128 Mainz, Germany

I. EVALUATING THE VOLUME FRACTION OF POLYSACCHARIDES AND WATER BY A VORONOI-MONTE CARLO CONSTRUCTION

The relative volume to be attributed to the polysaccharide and to the water fraction in each sample has been estimated using a Voronoi-type definition of the volume attributed to the polysaccharide and to water, coupled to a MC integration. In this procedure, a relatively large number of points N_r are distributed at random over the entire (cubic) system volume. Then, for each point, the closest atom is identified, and the random point is attributed to the species (i.e., polysaccharide or water) to which the atom belongs. The ratio of the number of polysaccharide- and water-points gives the required estimate of the volumes' ratio. Since the set of points closest to each atoms represents, by definition, the Voronoi cell associated to that atom, the procedure is equivalent to estimate the Voronoi volume attributed to the two species using Monte Carlo integration.

In the present case, $N_r = 10^5$ points have been used for each sample, giving an accuracy of the order of 1 %.

II. WATER OXYGEN (OW) STRUCTURE FACTORS IN CALLOSE / WATER, CELLULOSE / WATER E BULK WATER

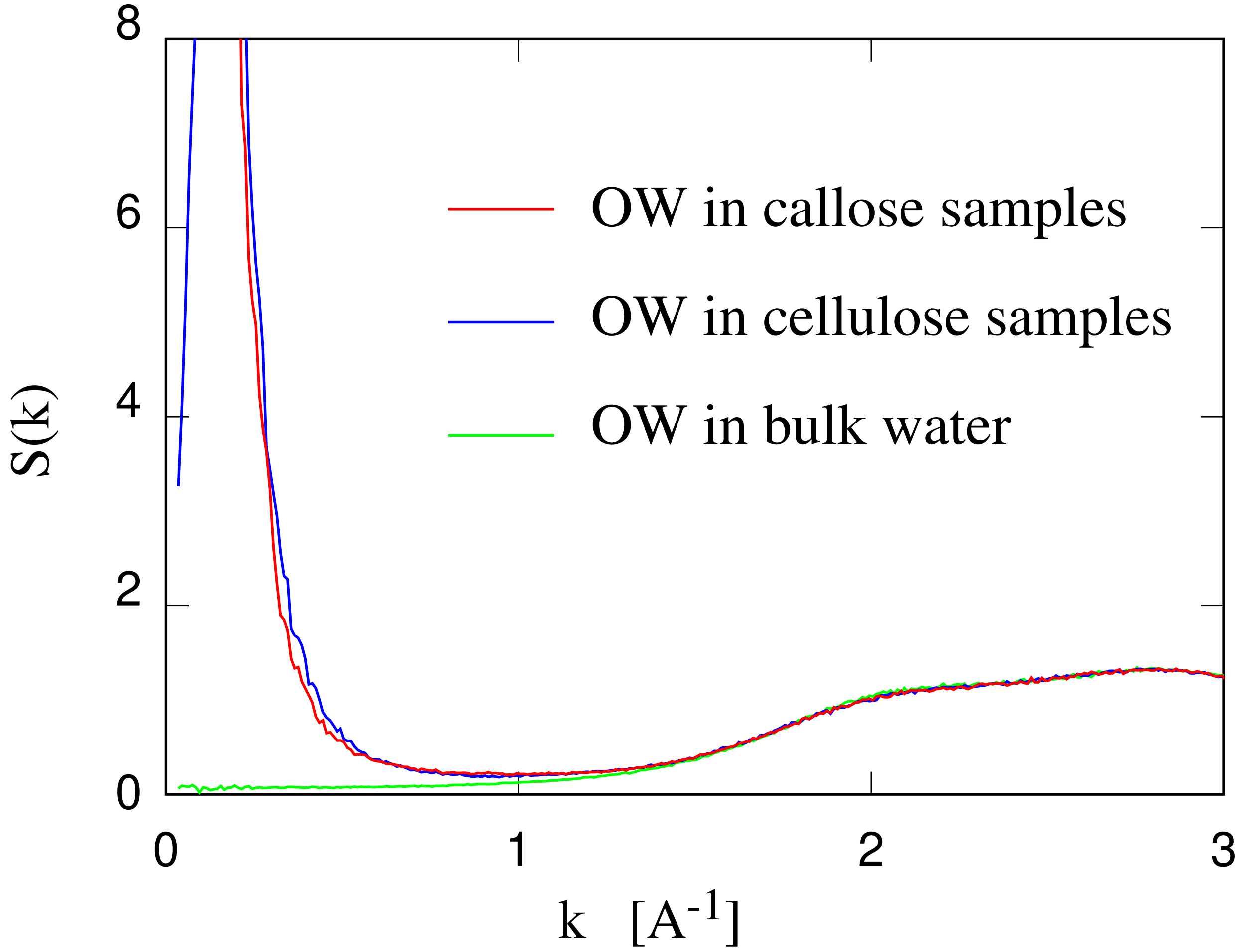

FIG. S1: Comparison of the structure factor computed for the water oxygen (OW) atoms in bulk water (green line), callose / water (red line) and cellulose /water (blue line) samples. Each sample contains $n_w = 18$ water molecules per glucane ring.

III. PRE-PEAK OF THE THE OXYGEN-OXYGEN STRUCTURE FACTORS IN POLYSACCHARIDE / WATER SAMPLES

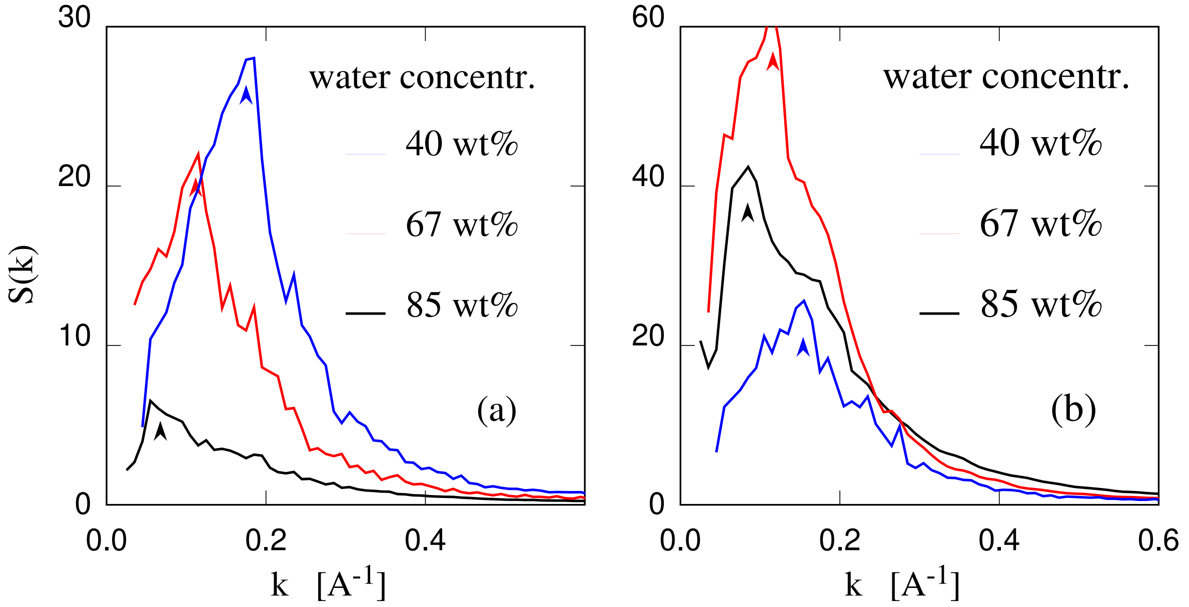

FIG. S2: Dependence of the pre-peak of the oxygen-oxygen structure factors $S(K)$ on the polysaccharide / water relative composition. Panel (a): water-water (OW-OW) structure factor. Panel (b): structure factor computed for the oxygen atoms belonging to the polysaccharide. The figure refers to callose / water samples; the results for cellulose / water show the same dependence of $S(k)$ on sample composition.

IV. DIFFUSION CONSTANT OF WATER IN MIXED CALLOSE-CELLULOSE / WATER SAMPLES

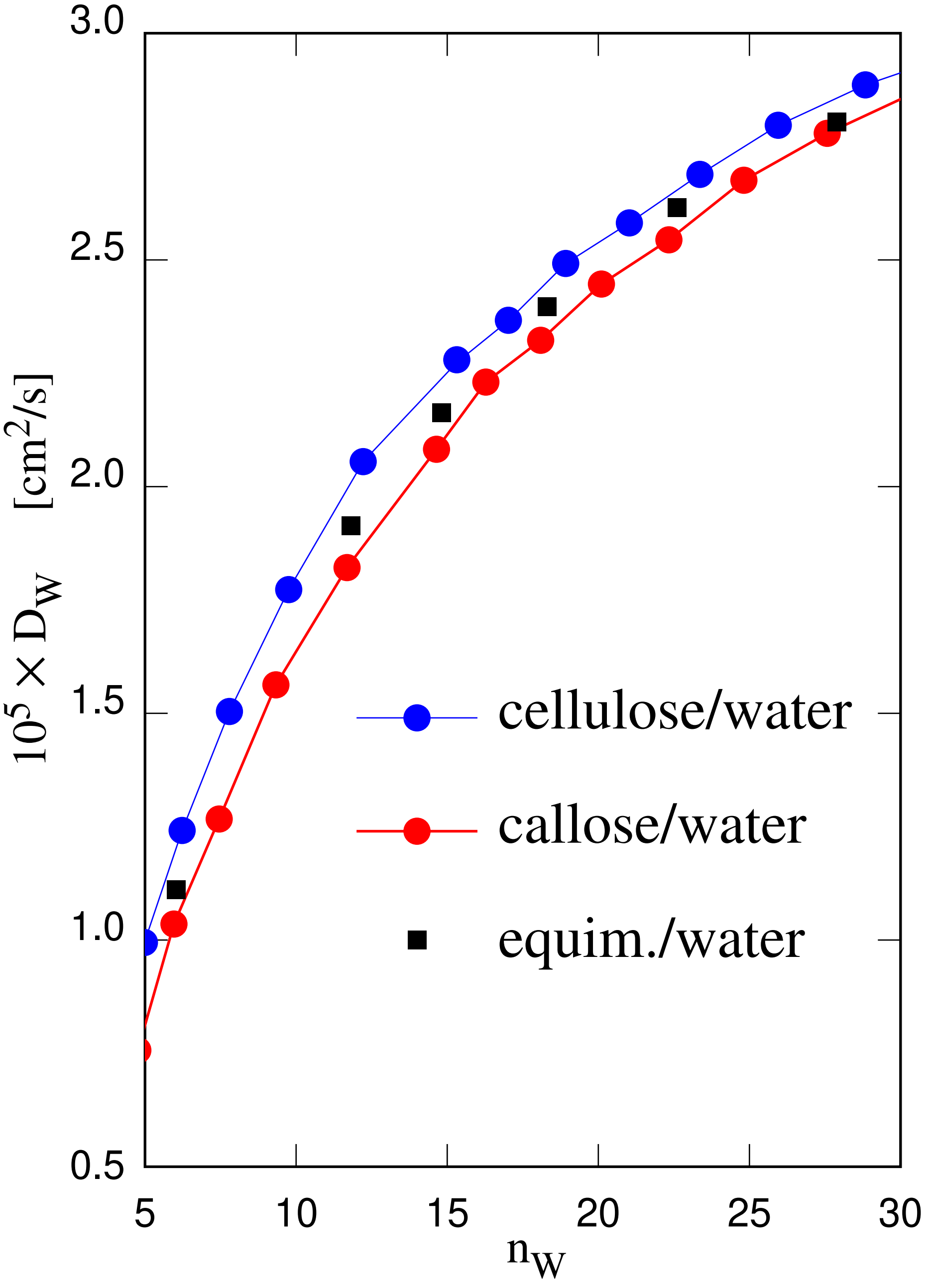

FIG. S3: Diffusion coefficient of water in equi-molar networks of callose and cellulose chains in water (black filled squares) as a function of water concentration. The corresponding data for the pure callose and cellulose chains in water are reported in red and blue, respectively, as a comparison. In all three cases, the size of the symbols has been scaled in order to correspond to the estimated error bar.

V. WATER DIFFUSION COEFFICIENT IN THE CALLOSE / WATER AND CELLULOSE / WATER SAMPLES

The water diffusion data illustrated in Fig. 5 of the main text (copied here in Fig. S3 (a)) have been reported on a new plot in this SI document (see Fig. S3 (b)) as a function of the number n_W of water molecules per glucose monomer in the sample. The two variables expressing water content, i.e., the water concentration in wt% and n_W are connected by a simple one to one relation, which however, is non-linear. For this reason, the second plot, shown in Fig. S4 (b), has a different appearance from panel (a), and emphasises aspects that are less apparent in the first representation. In particular, one can see that over the 18-85 wt% water concentration range, the water diffusion coefficient is a convex function of n_w, meaning that the entire curve is below the tangent at any of its points. Seen in reverse, this means that with increasing polysaccharide content, the slowing down of the water mobility is faster than linear.

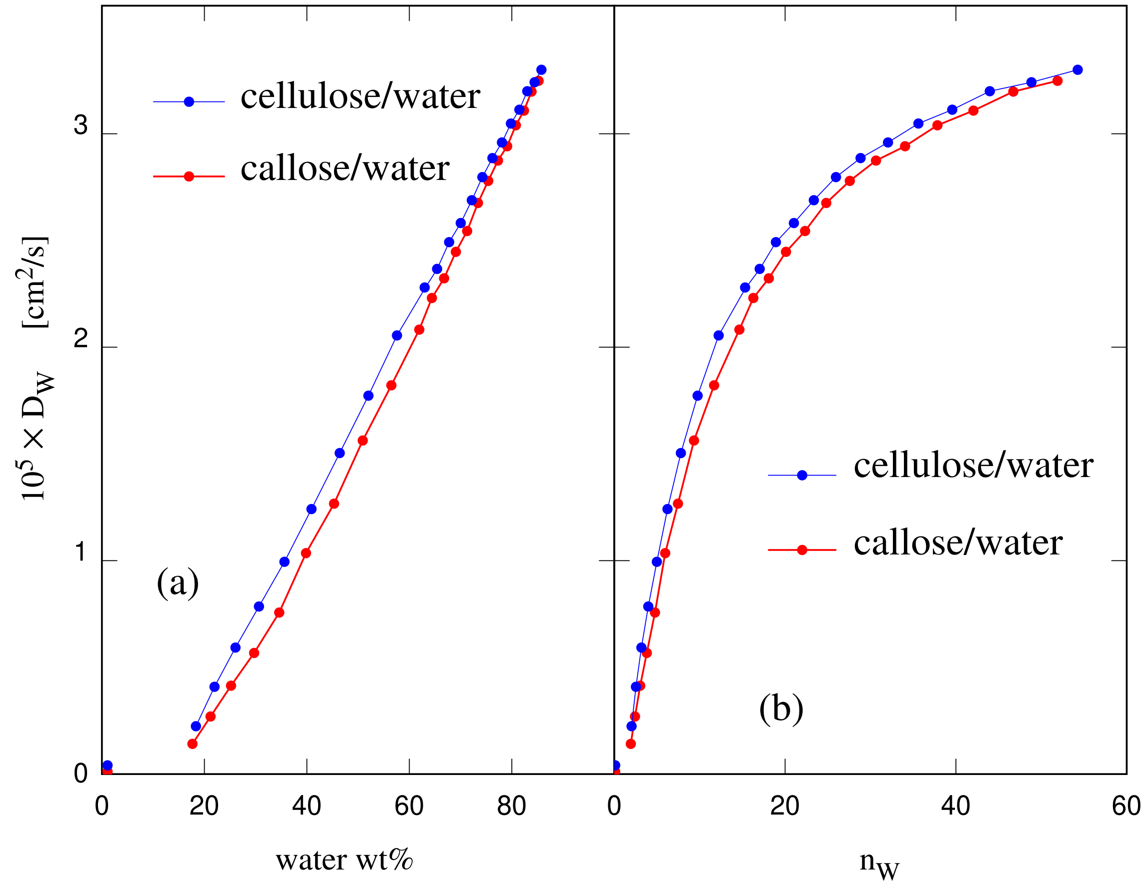

FIG. S4: (a) Water diffusion constant D_W as a function of water concentration (in wt%) in the polysaccharide/water samples; (b) same D_W as a function of the number of water molecules per glucose ring n_W. The error bar on each D_W value is comparable to the radius of the dots. The continuous lines are a guide to the eye. The red and blue dots close to the origin and not connected by the continuous lines represent two samples of very low water content (1 wt% water).

Test simulations at much lower water concentration (922 water molecules in $\sim$ 1850 nm^3, corresponding to $n_W = 0.1$, and representing 1 wt% water concentration) show that the motion of water remains diffusive down to the lowest water concentrations (see Fig. S5 below), with a residual D_W of water molecules in the nearly dry callose of $D_W = (1.2 \pm 0.3)\ 10^{-7}$ cm^2/s. At this concentration, water molecules are expected to diffuse independently from each other, while interacting strongly with the callose amorphous substrate. The

residual D_W of water in cellulose / water samples of the same relative concentration is $D_W = (2.1 \pm 0.3)\ 10^{-7}$ cm^2/s. These two values for callose and cellulose samples have been added in Fig. S4 (a) and (b), where they appear as isolated points close to the origin. On the S4 (a) panel, the two points are apparently out of line with the points joined by the continuos line, confirming that a change of diffusional regime takes place at low water concentration. On the other hand, the anomaly virtually disappears upon changing the variable that represents water concentration from wt% to n_W. Needless to say, the two plots describe the same system and the same properties, but adopting the n_W variable seems to result in a simpler interpolation of properties related to water mobility and overall system fluidity as a function of water content.

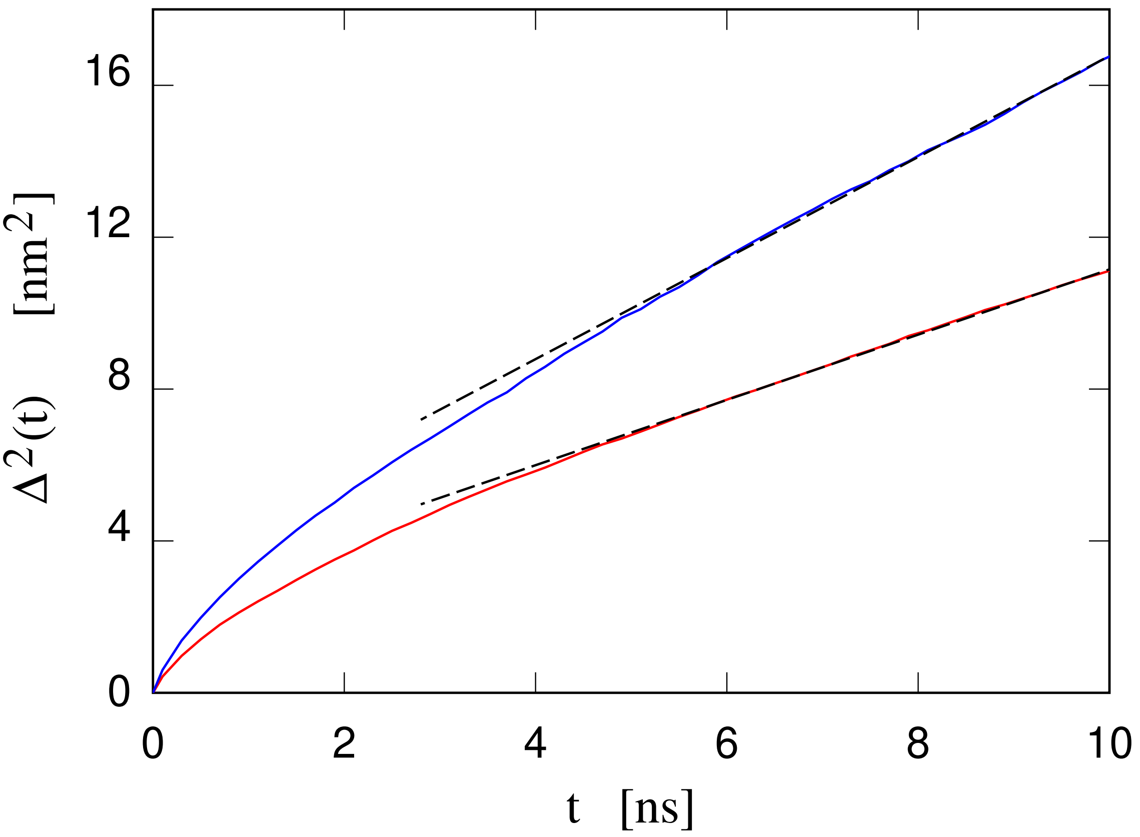

FIG. S5: Average square displacement per particle $\Delta^2(t)$ as a function of time of water oxygen atoms in callose / water (red line) or cellulose / water (blue line) samples of low water concentration ($n_w = 2$). The dash lines (black) are the linear interpolations to the two curves for $t \geq 5$ ns.

The average square displacement as a function of time is defined as

$$\Delta^2(t) = \langle| \mathbf{r_i}(t + t_0) - \mathbf{r_i}(t_0) |^2\rangle_{i,t_0} \tag{1}$$

The average implied in $\Delta^2(t)$ is over particles (i) and initial time t_0.

VI. FIT OF THE PROBABILITY DISTRIBUTION FOR THE DISPLACEMENT OF WATER MOLECULES OVER 30 PS

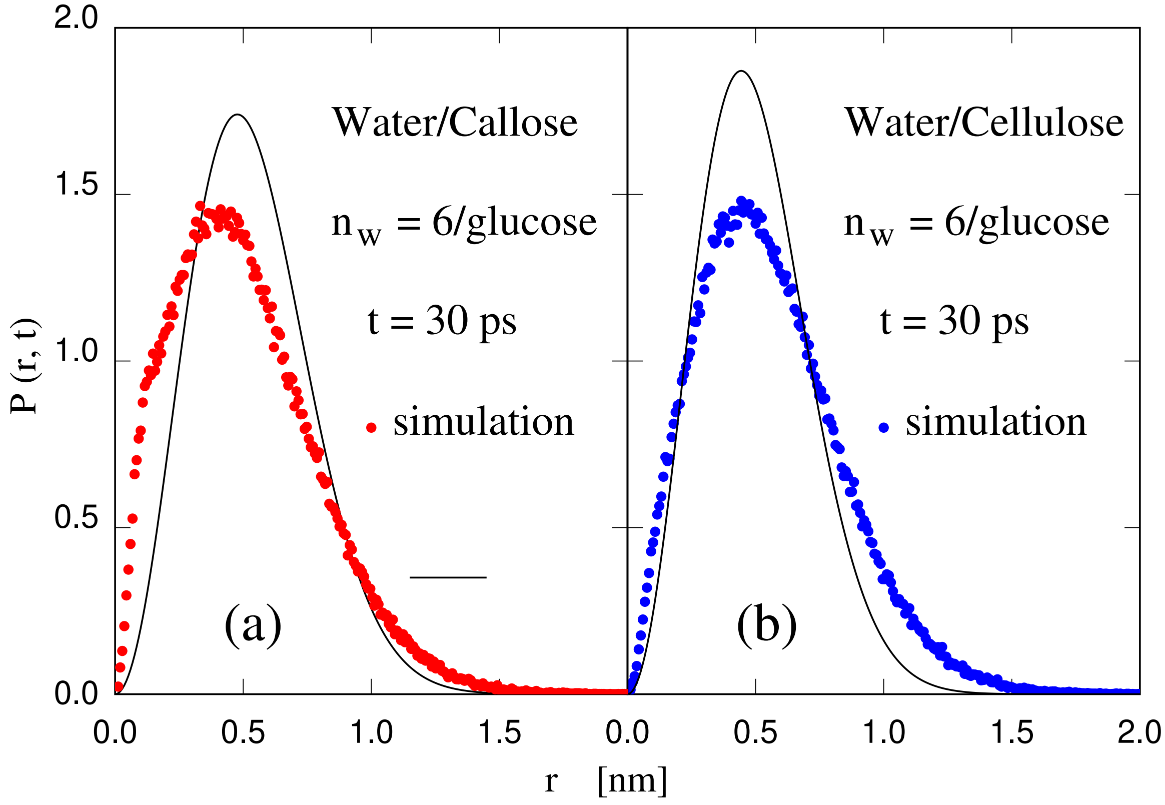

FIG. S6: Probability distribution $P(r,t)$ for the displacement r of water oxygen atoms over the time $t = 30$ ps. Dots: simulation results; full line: interpolation by a single Brownian distribution function (Eq. 3 of main text). Left panel: callose / water sample; right panel: cellulose / water sample. In both cases, the distribution from the fit is broader than the one from simulation. In the callose case, the discrepancy manifests itself mainly on the low-displacement side of the distribution, while the opposite is found for the cellulose / water data.

TABLE S1: Average number of intra-chain and inter-chain H-Bonds per glucane ring in callose / water and cellulose / water systems as a function of the number n_w of water molecules, also per glucose ring.

n_w	28	22	18	15	12	6
Callose/water						
$\langle n_{HB}(intra) \rangle$	0.63	0.66	0.67	0.67	0.68	0.71
$\langle n_{HB}(inter) \rangle$	0.12	0.14	0.15	0.16	0.17	0.21
Cellulose/water						
$\langle n_{HB}(intra) \rangle$	0.76	0.77	0.78	0.80	0.81	0.83
$\langle n_{HB}(inter) \rangle$	0.10	0.12	0.15	0.15	0.16	0.19

VII. RADIAL DISTRIBUTION FUNCTION OF WATER OXYGEN ATOMS.

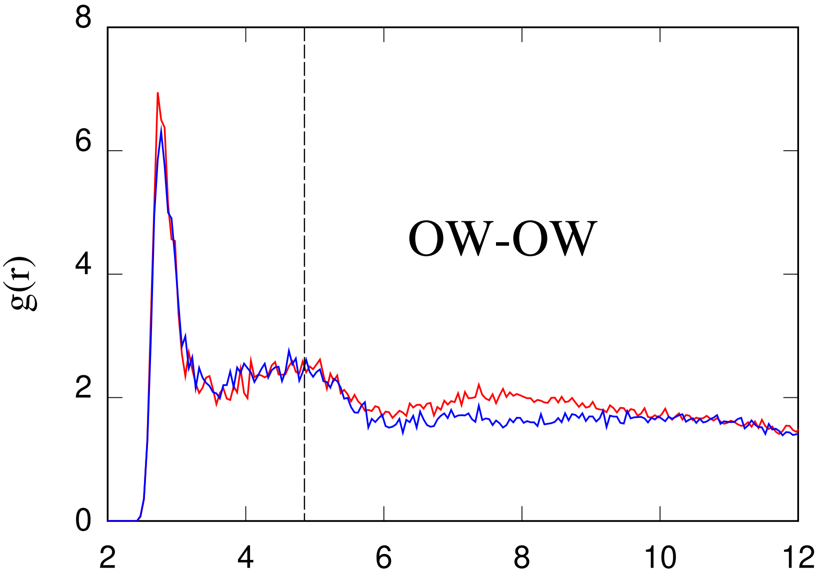

FIG. S7: Radial distribution function computed among the oxygen atoms belonging to water molecules H-bonded to callose (red line) or cellulose (blue line) chains.
